# Automated Segmentation of Digital Artifacts in Intraoral Photostimulable Phosphor Radiographs

**DOI:** 10.3390/diagnostics16081194

**Published:** 2026-04-16

**Authors:** Ceyda Gizem Topal, Osman Yalçın, Hatice Tetik, Murat Ünal, Necla Bandirmali Erturk, Cemile Özlem Üçok

**Affiliations:** 1 Department of Oral and Maxillofacial Radiology, Faculty of Dentistry, Yozgat Bozok University, 66000 Yozgat, Turkey; osman.yalcin@bozok.edu.tr; 2 Izmir Dental Hospital, 35250 Izmir, Turkey; 3 Department of Endodontics, Faculty of Dentistry, Yozgat Bozok University, 66000 Yozgat, Turkey; murat.unal@bozok.edu.tr; 4 Department of Computer Engineering, Faculty of Engineering and Natural Sciences, Bandirma Onyedi Eylul University, 10200 Balıkesir, Turkey; nerturk@bandirma.edu.tr; 5 Department of Oral and Maxillofacial Radiology, Faculty of Dentistry, Gazi University, 06490 Ankara, Turkey; ozlemucok@gazi.edu.tr

**Keywords:** artifact detection, deep learning, image quality, intraoral radiography, patient safety, photostimulable phosphor (PSP), quality assurance, SDG 3: good health and well-being

## Abstract

**Background/Objectives**: Intraoral radiographs acquired using photostimulable phosphor (PSP) plates are inherently susceptible to a wide spectrum of artifacts that can compromise diagnostic reliability and lead to unnecessary repeat exposures. Although structured taxonomies describing these artifacts have been proposed, automated methods capable of detecting and localizing multiple artifact types at the pixel level remain limited, particularly under realistic multi-class conditions. In this study, we address the problem of fine-grained, multi-class PSP artifact segmentation by systematically evaluating a deep learning-based framework and establishing a realistic baseline for this inherently challenging task. **Methods**: A retrospective, multi-center dataset comprising 1497 intraoral PSP radiographs (bitewing and periapical) collected from three institutions was analyzed. Pixel-level annotations were generated by expert oral and maxillofacial radiologists according to a standardized taxonomy consisting of four major artifact groups and 29 artifact classes, together with a background class. A 2D nnU-Net v2 architecture was employed as a baseline segmentation model. Model development was performed using 5-fold cross-validation, and performance was evaluated on an independent test set using Dice coefficient, Intersection over Union (IoU), Precision, and Recall. **Results**: Across all classes, the model achieved a mean Dice score of 0.0894 ± 0.0084 in cross-validation and 0.0952 on the independent test set, reflecting the intrinsic complexity of the task. Class-wise analysis revealed substantial variability, with higher performance in larger and visually distinctive artifacts, whereas small-scale, low-contrast, and underrepresented classes exhibited markedly reduced performance. Notably, several artifact categories were absent from the training data, resulting in a zero-shot scenario that directly constrained model generalization. Furthermore, segmentation performance demonstrated a strong dependency on class frequency, measured in terms of pixel distribution, underscoring the impact of severe class imbalance. Group-based evaluation showed relatively higher performance for pre-exposure and exposure-related artifacts compared to post-exposure and scanner-related categories. **Conclusions**: These findings demonstrate that large-scale, multi-class pixel-level segmentation of PSP artifacts represents a fundamentally challenging problem shaped by the combined effects of class imbalance, small object size, heterogeneous artifact morphology, and incomplete training representation. While the proposed framework confirms the feasibility of automated artifact localization, its current performance suggests greater immediate value as a quality control or screening support tool rather than a fully autonomous diagnostic system. By providing a comprehensive baseline and systematic analysis, this study establishes a benchmark for future research and highlights the critical need for imbalance-aware learning strategies, hierarchical modeling, and data-centric approaches to advance this field.

## 1. Introduction

Intraoral radiographs constitute a fundamental component of diagnostic decision-making in dentistry; however, their clinical value is directly dependent on image quality. The presence of imaging artifacts may reduce diagnostic reliability, lead to repeat examinations, and increase unnecessary radiation exposure [1,2]. This issue becomes particularly critical in clinical environments with high patient turnover, where maintaining consistent image quality and ensuring patient safety remain significant challenges [3,4].

Photostimulable phosphor (PSP)-based digital systems are widely used in intraoral radiography due to their wireless design, ease of use, and low radiation dose requirements [5,6]. However, both the physical properties of PSP plates and the scanning process make these systems prone to a wide range of artifacts [7,8]. Positioning errors, improper placement of the phosphor plate, contamination, and scanner-related technical issues are among the most common causes of artifacts [9,10]. Previous studies have proposed structured taxonomies that categorize these artifacts according to their origin, such as pre-exposure, exposure-related, post-exposure, and scanner-related [11].

In routine clinical practice, artifact detection is generally performed after image acquisition and largely depends on operator experience. This process is inherently subjective and may lead to variability in quality assessment, particularly in settings where radiographs are acquired by students or technicians [3,4,12]. Moreover, due to the high clinical workload, it is often not feasible for radiology specialists to review all images immediately after acquisition [2,4].

Quantitative evidence further supports these limitations, as reported mean rejection rates in intraoral radiography reach 11.25% for bitewing and 16.38% for periapical radiographs. In this context, ‘rejection rate’ is defined as the proportion of radiographs deemed diagnostically unacceptable—and thus requiring a retake—due to technical or patient-related failures. Based on established taxonomies, these artifacts are primarily categorized into operator errors (e.g., positioning errors and cone cuts), plate errors (e.g., bite marks and scratches), and scanning errors (e.g., delayed scanning or line artifacts). Specifically, periapical radiographs demonstrate higher rejection rates (up to 80.8% of all retakes) compared to bitewings. This disparity is attributed to the discomfort and gagging caused by placing the receptor deep into the oral cavity to capture root apices, a factor that frequently leads to positioning failures. These findings underscore that current manual quality control strategies are insufficient to effectively prevent unnecessary radiation burden and highlight the need for automated detection frameworks [13,14].

These limitations highlight the need for automated systems capable of detecting artifact-containing intraoral radiographs at an early stage in an objective and operator-independent manner. In recent years, deep learning-based approaches have demonstrated strong performance in various dental imaging tasks, including caries detection, identification of periapical lesions, and segmentation of anatomical structures [15,16,17]. However, existing studies have largely focused on diagnostic targets, while the automated detection and localization of artifacts that directly affect image quality remain relatively underexplored [18].

At the same time, the analysis of PSP-related artifacts presents several inherent challenges. These artifacts exhibit considerable morphological diversity, vary substantially in size, and are often confined to small regions within the image. In addition, the distribution of artifact classes is typically highly imbalanced, and some artifact categories may be absent from the training data, leading to a zero-shot scenario. Taken together, these characteristics make fine-grained multi-class segmentation of dental artifacts substantially more difficult than many conventional medical image segmentation problems.

Recent advances in medical image analysis have been driven by self-configuring deep learning frameworks, particularly nnU-Net (German Cancer Research Center (DKFZ), Heidelberg, Germany), which has shown robust performance across diverse segmentation tasks by automatically adapting preprocessing, architecture, and training strategies to the characteristics of the dataset [18,19,20,21,22].

In this study, we address the problem of fine-grained, multi-class PSP artifact segmentation by developing and systematically evaluating a deep learning-based framework. Rather than focusing solely on performance optimization, the present study aims to establish a realistic and reproducible baseline under challenging real-world conditions, including severe class imbalance and the presence of zero-shot artifact categories. In addition to addressing this gap, the study contributes to the literature in several important respects. First, it introduces a multi-center dataset for fine-grained PSP artifact segmentation, comprising 29 artifact classes with expert-annotated pixel-level masks. Second, it provides a comprehensive benchmark evaluation of deep learning-based segmentation under challenging real-world conditions characterized by class imbalance, small artifact structures, and missing training categories. Third, it systematically examines the relationship between artifact characteristics, such as size and class frequency, and segmentation performance. Taken together, these contributions provide both a practical baseline and a clearer understanding of the challenges of automated artifact detection in dental radiography.

## 2. Materials and Methods

### 2.1. Study Design and Dataset

This study was designed as a retrospective, multi-center diagnostic image analysis study. A total of 1497 intraoral PSP radiographs were collected from three institutions: Gazi University Faculty of Dentistry, Yozgat Bozok University Faculty of Dentistry, and Izmir Dental Hospital. All radiographic images were anonymized prior to analysis to ensure patient confidentiality. The inclusion of data from multiple centers was intended to increase dataset diversity and improve the robustness of the analysis. Ethical approval for the study was obtained from the Ethics Committee of Gazi University (Approval No.: 2022-897).

### 2.2. Imaging Protocol and Technical Specifications

All radiographs were acquired using a Planmeca Pro X™ 2D intraoral X-ray unit (Planmeca^®^, Helsinki, Finland) with size 2 PSP plates from the same manufacturer. Standard exposure parameters (65 kVp, 7 mA, 0.2 s) were applied across all participating centers to ensure consistency in image acquisition.

For artificial intelligence-based analysis, all images were resampled to a uniform spatial resolution of 629 × 799 pixels and stored in JPEG format using standard export settings. This standardization step was applied to ensure consistent input dimensions for the deep learning model. No additional compression or post-processing was applied in order to preserve the original clinical characteristics of the images. Inclusion criteria comprised intraoral PSP radiographs with sufficient diagnostic quality and clearly identifiable artifact structures suitable for annotation. Exclusion criteria included images with severe geometric distortion, incomplete anatomical coverage, or inadequate image quality that could compromise reliable artifact assessment.

### 2.3. Artifact Annotation and Taxonomy

Artifact annotations were performed using the Computer Vision Annotation Tool (CVAT; an open-source platform initially developed by Intel and currently maintained by CVAT.ai; available at https://www.cvat.ai/ (accessed on 6 February 2026)). Each radiograph was manually annotated at the pixel level by three experienced oral and maxillofacial radiologists with expertise in PSP artifact interpretation and 8, 7, and 5 years of clinical experience, respectively. The annotation process was not performed under blinded conditions, as the aim was consensus-based expert delineation rather than inter-observer comparison. Polygon-based segmentation masks were generated to delineate artifact regions according to a predefined taxonomy. Artifacts were categorized into four main groups based on the classification proposed by Incebeyaz et al. [11]: (G1) pre-exposure artifacts (e.g., phosphor plate scratches and physical damage), (G2) exposure-related artifacts (e.g., positioning errors, motion blur, and cone-cut), (G3) post-exposure artifacts (e.g., processing-related noise and fading), and (G4) scanner-related artifacts (e.g., plate misalignment and line artifacts).

Each annotated artifact instance was assigned to one of 29 classes, along with a background class. This fine-grained labeling scheme was designed to enable detailed analysis of artifact types; however, it also introduced increased task complexity and class imbalance.

To ensure annotation consistency, all annotations were independently performed and subsequently reviewed. Discrepancies between annotators were resolved through consensus, resulting in a unified ground truth for each image. This consensus-based annotation approach is commonly adopted in medical image analysis to reduce inter-observer variability.

The final dataset consisted of pixel-wise labeled segmentation masks, which were used as ground truth for both model training and evaluation.

### 2.4. Deep Learning Framework

**Figure 1:**
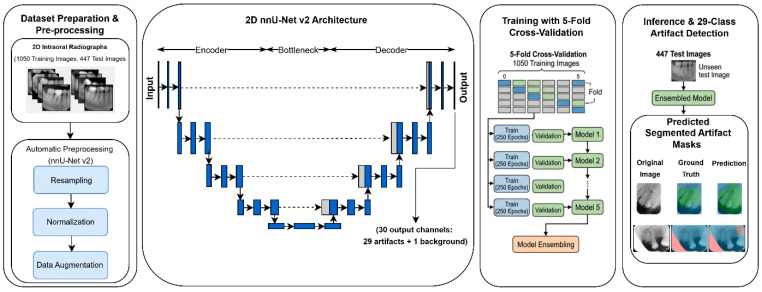
Proposed nnU-Net v2 architecture and training pipeline for 29 artifact detection in 2D intraoral radiographic dental images.

A 2D nnU-Net v2 framework was employed for pixel-level artifact segmentation of PSP-related artifacts (Figure 1). All experiments were conducted using nnU-Net v2 (version 2.6.4). nnU-Net is a self-configuring deep learning framework that automatically adapts preprocessing steps, network architecture, and training parameters to the specific characteristics of the dataset, including target spacing, patch size, batch size, and intensity normalization strategies.

In this study, nnU-Net v2 was used as a standardized and reproducible baseline model, allowing the evaluation of segmentation performance under challenging conditions without extensive manual optimization. This approach enables a fair assessment of the intrinsic difficulty of the task, particularly in the presence of severe class imbalance and fine-grained artifact categories. This design choice allows the results to be interpreted as a realistic benchmark rather than as an optimized upper-bound estimate of performance.

The model is based on a U-Net-like-based encoder–decoder architecture with skip connections, which facilitates the extraction of both global contextual features and fine-grained spatial details. The network was trained using a compound loss function combining Dice loss and cross-entropy loss, balancing region overlap accuracy and pixel-wise classification performance.

Optimization was performed using the Adam optimizer with an adaptive learning rate, as defined within the nnU-Net training framework. All training configurations followed the default nnU-Net v2 pipeline to ensure consistency and comparability with existing studies.

### 2.5. Dataset Preparation and Splitting

The dataset was organized according to the standardized nnU-Net format, where each radiograph was paired with its corresponding pixel-wise segmentation mask.

A total of 1497 intraoral PSP radiographs were included in the study. Of these, 1050 images were allocated to the model development set. Within this set, a 5-fold cross-validation strategy was employed, where the data were iteratively partitioned into training and validation subsets to ensure robust and reliable performance estimation.

To evaluate model generalizability, an independent test set consisting of 447 previously unseen images was used. These images were strictly excluded from all training and validation processes. Data splitting was performed on a per-patient basis to prevent data leakage between training, validation, and test sets. This evaluation protocol ensures both robust internal validation and an unbiased assessment of model performance on unseen data.

### 2.6. Preprocessing and Data Augmentation

During preprocessing, images were standardized in spatial resolution, and intensity normalization was applied to ensure consistent input distributions across the dataset. Foreground cropping was performed to remove irrelevant background regions and focus the model on anatomically relevant areas.

Preprocessing and data augmentation were conducted using the default nnU-Net v2 pipeline. Online data augmentation techniques, including spatial transformations and intensity variations, were applied during training to improve model generalizability and robustness.

The dataset exhibited substantial class imbalance, with certain artifact classes being highly represented while others had limited or no representation. No explicit class balancing strategy (e.g., class weighting or oversampling) was applied during training. This decision was made intentionally to preserve the natural distribution of the dataset and to evaluate model performance under realistic conditions. This setting allows the results to be interpreted as a baseline performance under real-world data conditions.

### 2.7. Training Procedure

Model training was performed using patch-based mini-batch learning within the nnU-Net v2 framework, enabling efficient learning of both local and contextual features from high-resolution radiographs. Image patches were automatically sampled by the nnU-Net framework during training, allowing the model to learn both local and contextual representations without manual patch definition.

A 5-fold cross-validation strategy was employed on the model development set to assess model robustness and reduce the risk of overfitting. In this setup, the dataset was partitioned into five folds, and the model was iteratively trained using different combinations of training and validation subsets.

For final evaluation, models obtained from each fold were combined in an ensemble manner during inference, as implemented in the nnU-Net v2 framework. Overall performance was reported as the mean and standard deviation across the five folds. This ensemble-based strategy improves prediction stability and supports generalization by aggregating outputs from multiple trained models.

### 2.8. Inference and Post-Processing

During inference, full-resolution images were processed using a sliding-window approach as implemented in the nnU-Net v2 framework. The trained model generated pixel-wise probability maps, which were converted into final segmentation masks using softmax and argmax operations.

Post-processing included removal of small isolated regions using connected component analysis. This step was applied to reduce spurious predictions and improve the consistency of the segmentation outputs, particularly for classes with limited spatial extent. No additional heuristic or class-specific post-processing was applied.

### 2.9. Evaluation Metrics

Segmentation performance was evaluated using pixel-wise comparisons between predicted and ground truth masks. The following metrics were calculated: Dice coefficient (F1-score), Intersection over Union (IoU), Precision, and Recall.

To account for the substantial class imbalance in the dataset, performance metrics were primarily reported using macro-averaging, where each class contributes equally regardless of its frequency. This approach provides a more balanced evaluation across both common and rare artifact classes. In addition to overall macro-averaged metrics, class-wise performance scores were also computed to enable detailed analysis of individual artifact categories.

All metric calculations and statistical analyses were performed using Python-based implementations (Python version 3.12; Python Software Foundation, Wilmington, DE, USA). Macro-averaged metrics were preferred as they provide a more reliable evaluation in the presence of classes with zero predictions. This study focuses exclusively on pixel-level segmentation performance. Although artifact classes are defined at the category level, no separate image-level classification task was explicitly evaluated. This study primarily reports descriptive performance statistics. No inferential statistical hypothesis testing was performed, as the objective was to provide a benchmark evaluation across folds and on the independent test set.

## 3. Results

### 3.1. Overall Model Performance

The overall segmentation performance of the proposed framework was modest across the diverse set of artifact classes. The Dice coefficient, equivalent to the F1-score in pixel-level segmentation, was used as the primary evaluation metric.

In the 5-fold cross-validation, the model achieved a macro-averaged Dice score of 0.0894 ± 0.0084. Evaluation on the independent test set yielded a comparable Dice score of 0.0952, indicating consistent generalization performance on unseen data.

Similarly, IoU, precision, and recall values remained low across both validation and test settings. These results reflect the inherent difficulty of fine-grained, multi-class segmentation involving 29 artifact categories in intraoral PSP radiographs, particularly under conditions of severe class imbalance, small artifact sizes, and the presence of underrepresented or absent classes in the training data.

Rather than indicating a limitation of the modeling framework itself, these findings highlight the complexity of the task under real-world data conditions and emphasize the need for more advanced, imbalance-aware learning strategies. The overall segmentation performance is summarized in Table 1.

### 3.2. Class-Wise Segmentation Performance

**Figure 2:**
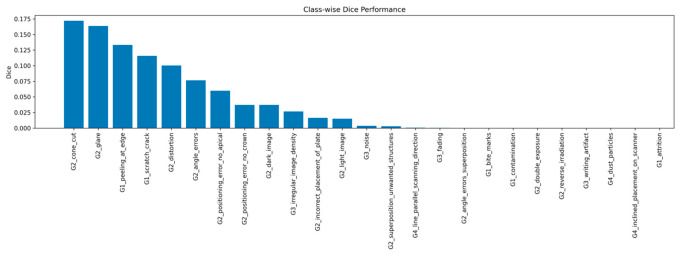
Class-wise Dice scores across the 29 PSP artifact categories, illustrating substantial variability in segmentation performance.

Segmentation performance varied substantially across artifact classes, indicating a highly heterogeneous learning behavior across the defined taxonomy (Figure 2). The highest Dice scores were observed in classes with distinct geometric features, including G2_cone_cut (≈0.42), G2_glare (≈0.30), G1_peeling_at_edge (≈0.22), and G1_scratch_crack (≈0.19). These relatively higher-performing categories correspond to artifacts that occupy larger spatial regions and exhibit clear visual boundaries, enabling the model to learn more discriminative representations.

In contrast, a considerable number of classes demonstrated near-zero Dice scores. These classes were generally associated with small, sparse, or low-contrast structures, such as dust particles and writing artifacts, or subtle intensity variations, such as fading. Moreover, several low-performing classes were either severely underrepresented or completely absent in the training data, resulting in a zero-shot scenario that inherently limited model performance for these categories.

This wide performance gap across classes highlights a strong dependence of segmentation accuracy on both artifact size and class frequency. A summary of the best- and worst-performing classes is presented in Table 2.

### 3.3. Relationship Between Artifact Size and Performance

**Figure 3:**
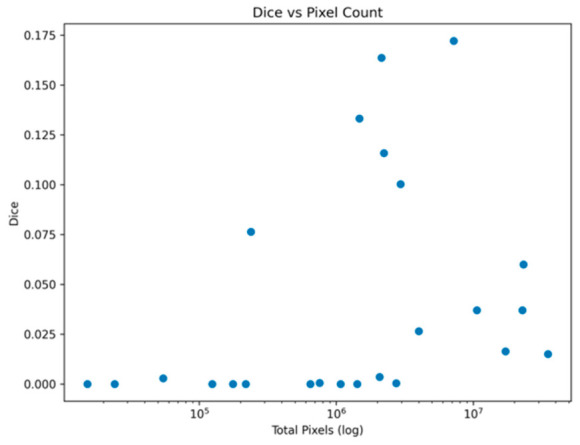
Relationship between artifact size and segmentation performance, showing higher Dice scores for artifact classes with greater pixel coverage.

A clear positive relationship was observed between artifact size (measured as total pixel count) and segmentation performance (Figure 3).

This trend indicates that the model is more effective at capturing spatially extensive and structurally consistent regions, which provide stronger contextual and boundary information during training. In contrast, fine-grained artifacts with limited spatial extent and low contrast present insufficient discriminative cues, leading to reduced segmentation accuracy.

Moreover, the observed pattern is consistent with the class-wise performance analysis, further supporting that artifact size is a key factor influencing model performance in this multi-class setting.

### 3.4. Group-Wise Analysis

**Figure 4:**
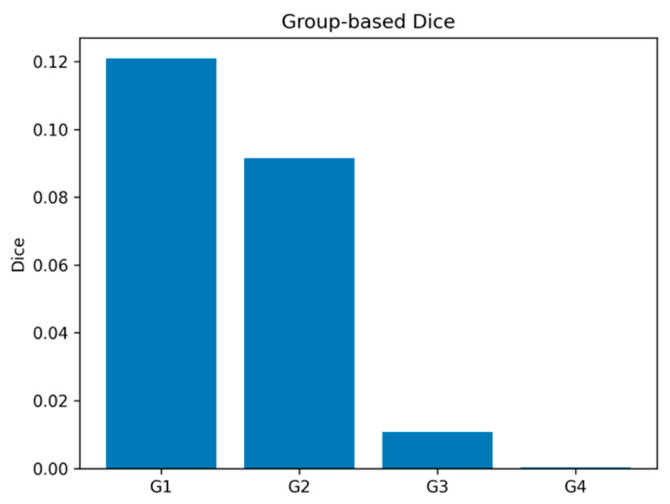
Group-wise segmentation performance across the four main PSP artifact categories, showing relatively higher performance in G1 and G2 than in G3 and G4.

When artifact classes were aggregated into the four main taxonomy groups, distinct performance differences emerged (Figure 4, Table 3). The Dice scores were approximately 0.1210 for G1 (pre-exposure artifacts), 0.0915 for G2 (exposure-related artifacts), 0.0108 for G3 (post-exposure artifacts), and 0.0003 for G4 (scanner-related artifacts).

The model demonstrated relatively higher performance in pre-exposure (G1) and selected exposure-related (G2) artifacts. These groups include artifact types that are generally more structured, visually distinct, and spatially extensive, making them more amenable to segmentation.

In contrast, performance dropped substantially for post-exposure (G3) and scanner-related (G4) artifacts. These categories typically consist of subtle, diffuse, or noise-like patterns, often characterized by low contrast and limited spatial consistency. In addition, several classes within these groups were underrepresented or absent in the training data, further contributing to the observed performance degradation.

Overall, the group-wise analysis reinforces that both artifact characteristics (e.g., size, contrast, and structural consistency) and data distribution play a critical role in determining segmentation performance.

### 3.5. Class Distribution and Imbalance

**Figure 5:**
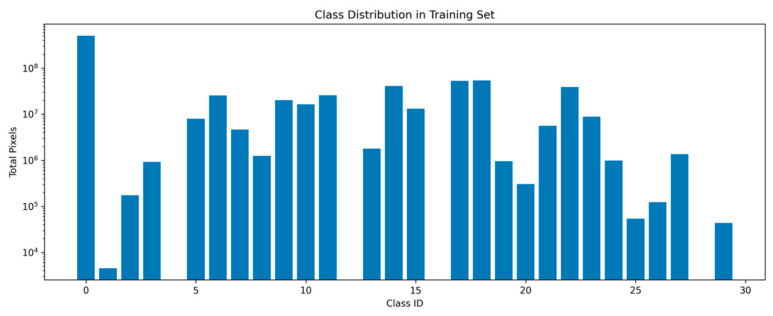
Distribution of artifact classes in the training dataset, illustrating pronounced imbalance in both class frequency and pixel coverage.

The training dataset exhibited a pronounced class imbalance across artifact categories, both in terms of pixel distribution and sample frequency (Figure 5). A small number of classes accounted for a large proportion of the total labeled pixels, whereas many artifact classes were sparsely represented.

Notably, several artifact categories had extremely limited pixel coverage, and a subset of classes was entirely absent from the training data. This resulted in a zero-shot scenario for these classes, where the model had no opportunity to learn representative features during training.

Such severe imbalance directly influenced the segmentation performance, as the model inherently favored dominant classes while failing to generalize to rare or unseen artifact types. This imbalance is consistent with the class-wise and group-wise performance analyses, where underrepresented classes exhibited near-zero Dice scores.

Overall, these findings highlight that class imbalance is a primary limiting factor in multi-class PSP artifact segmentation and must be explicitly addressed in future work through data-centric and imbalance-aware learning strategies.

### 3.6. Qualitative Evaluation

**Figure 6:**
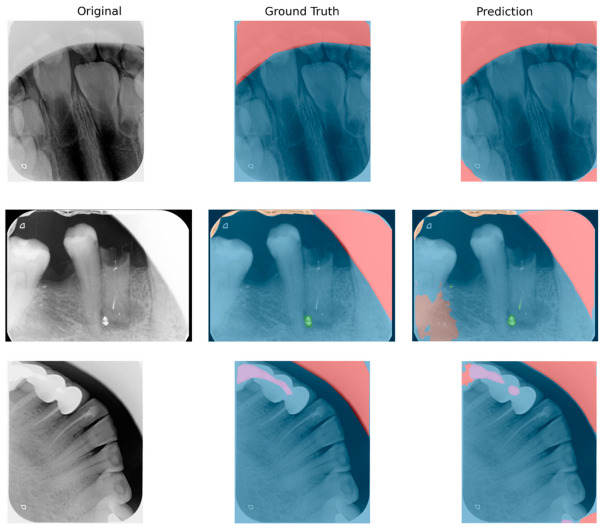
Representative qualitative examples of segmentation results. Each row represents a different case. From left to right: original intraoral PSP radiograph, ground truth annotation, and model prediction. The colored regions indicate segmented artifact areas, with different colors corresponding to different artifact classes. The examples illustrate both successful segmentation in prominent artifact regions and typical failure modes, including over-segmentation, false positives, and missed detections.

Representative qualitative examples of segmentation outputs are presented in Figure 6. The model demonstrated reasonable agreement with ground truth in artifact regions that were large, well-defined, and characterized by clear boundaries. However, notable discrepancies were observed in smaller, low-contrast, or structurally ambiguous artifact regions. In such cases, the model frequently produced over-segmentation in anatomically complex areas, false-positive predictions in regions with similar intensity patterns, and missed detections for subtle or sparsely distributed artifacts.

These qualitative observations are consistent with the quantitative results and further confirm that segmentation performance is strongly influenced by artifact size, visual distinctiveness, and class representation in the training data.

## 4. Discussion

The diagnostic reliability of intraoral radiography depends strongly on image quality. However, the widespread use of photostimulable phosphor (PSP) systems has introduced a diverse range of artifacts that may compromise image interpretation [11]. Due to their physical sensitivity and susceptibility to handling and scanning-related errors, PSP plates present persistent challenges for quality assurance in routine clinical workflows [8].

In current clinical practice, image quality assessment largely relies on manual visual inspection, which is subjective and operator-dependent. In this context, rejection rate refers to the proportion of radiographs considered diagnostically unacceptable and therefore requiring retake in the cited studies. Reported rejection rates for intraoral radiographs may reach mean values of up to 16.38%, leading to unnecessary repeat exposures and increased radiation burden [13]. These limitations underscore the need for automated, objective, and standardized quality assessment systems.

Most artificial intelligence-based studies in dental radiology have focused primarily on diagnostic tasks, such as caries detection, periapical lesion identification, and endodontic assessment, often reporting high performance. High performance has been reported for tasks such as caries detection, periapical lesion identification, and endodontic assessment [17,18,23]. However, these approaches generally assume that input images are of adequate quality. In practice, artifacts such as scratches, scanner-related distortions, or motion blur can significantly alter image characteristics and negatively affect the reliability of downstream diagnostic models. Therefore, artifact detection should be considered critical preliminary step in the radiographic analysis pipeline.

The present study addresses this gap by proposing a deep learning-based segmentation framework for automated PSP artifact detection. The nnU-Net architecture was selected due to its self-configuring design, which adapts preprocessing, network architecture, and training parameters to the dataset characteristics [19]. Despite these advantages, the overall segmentation performance remained limited. The model achieved Dice scores of 0.0894 ± 0.0084 in cross-validation and 0.0952 on the independent test set, with similarly low values observed for IoU, precision, and recall. Rather than indicating a limitation of the modeling framework itself, these results should be interpreted in the context of the inherent complexity of the task.

Specifically, the combination of a high number of artifact classes (*n* = 29), severe class imbalance, and the presence of underrepresented or entirely absent classes in the training data created a highly challenging learning scenario. In particular, the existence of zero-shot classes, artifact categories not observed during training, directly limited the model’s ability to generalize to these classes.

Class-wise analysis revealed that larger and visually distinct artifacts, such as cone-cut and glare, were segmented with relatively higher accuracy, whereas small, low-contrast, or sparsely distributed artifacts exhibited near-zero performance. This observation is consistent with the demonstrated relationship between artifact size and segmentation performance, as well as with prior studies in medical image segmentation, which report degraded performance for small objects and rare classes due to limited pixel representation and increased sensitivity to noise [24].

Group-wise evaluation further supported these findings. The model performed relatively better in pre-exposure (G1) and exposure-related (G2) artifacts, while performance for post-exposure (G3) and scanner-related (G4) artifacts remained extremely limited. These differences can be attributed to variations in structural characteristics, contrast, and spatial consistency across artifact types.

In particular, scanner-related artifacts often appear as thin, high-frequency linear patterns, which are prone to information loss during downsampling operations in convolutional neural networks [19,25,26]. This further complicates their accurate segmentation.

A key strength of this study is the use of a multi-center dataset, which enhances the robustness and generalizability of the findings. This strengthens the robustness of the findings and supports the potential applicability of the model in diverse clinical settings. Compared with single-center studies, multi-center datasets introduce greater variability in acquisition conditions and imaging characteristics, thereby providing a more realistic evaluation setting [27,28,29]. Furthermore, ground truth annotations were generated through consensus labeling by experienced oral and maxillofacial radiologists using the CVAT platform, which improves annotation reliability and consistency [30].

Importantly, rather than optimizing model performance under controlled conditions, this study aims to establish a realistic baseline and benchmark for multi-class PSP artifact segmentation. The findings highlight fundamental challenges associated with this task and provide a foundation for future research focusing on imbalance-aware learning strategies, hierarchical modeling, and data-centric approaches. At its current stage, the proposed system is more appropriately positioned as a quality control and screening support tool than as a fully autonomous diagnostic system.

### Limitations

This study has several limitations that should be considered when interpreting the findings. The most important limitation is the severe class imbalance across the 29 artifact categories. Several classes were represented by a very limited number of pixels, and some were entirely absent from the training data, resulting in a zero-shot scenario. This significantly constrained the model’s ability to learn robust and generalizable feature representations for rare artifact types.

Segmentation performance was also limited for small-scale or low-contrast artifacts, such as dust particles or fading. These structures are particularly challenging for deep learning models, as fine-grained details may be partially lost during downsampling operations within convolutional architectures.

In addition, the complex anatomical structure of intraoral radiographs introduces substantial visual overlap between anatomical features and artifact patterns. This overlap occasionally led to false-positive predictions and increased the difficulty of accurately distinguishing artifacts from normal anatomical structures.

Finally, although a multi-center dataset was used, all radiographs were acquired using a single imaging system. This may limit the generalizability of the findings to other devices with different imaging characteristics and acquisition protocols.

Despite these limitations, the study provides a realistic and comprehensive evaluation of multi-class PSP artifact segmentation under challenging and clinically relevant conditions.

## 5. Conclusions

This study demonstrates the feasibility of a baseline deep learning approach for automated detection and pixel-level segmentation of PSP-related artifacts in intraoral radiographs. By employing a comprehensive 29-class taxonomy and a multi-center dataset, the proposed nnU-Net-based approach establishes a realistic baseline for multi-class artifact segmentation under clinically relevant conditions.

Although the overall segmentation performance remains limited, the findings clearly reveal the intrinsic challenges of this task, particularly the effects of severe class imbalance, small artifact sizes, and the presence of underrepresented or zero-shot classes. These results indicate that multi-class PSP artifact segmentation is a highly complex problem that requires more than conventional modeling approaches.

From a clinical perspective, the early and automated identification of artifact-prone radiographs has the potential to improve image quality control, reduce unnecessary repeat exposures, and enhance workflow efficiency in dental radiology.

Future research should focus on imbalance-aware learning strategies, hierarchical or multi-stage modeling approaches, and data-centric improvements, including more balanced datasets and increased representation of rare artifact classes. Such developments will be essential for achieving clinically reliable performance and enabling the integration of automated quality assessment systems into routine practice.

## Figures and Tables

**Table 1:** Overall segmentation performance.

Metric	Cross-Validation (Mean ± SD)	Test Set
Dice	0.0894 ± 0.0084	0.0952
IoU	0.0664 ± 0.0067	0.0706
Precision	0.1493 ± 0.0154	0.1707
Recall	0.2894 ± 0.0209	0.2601

**Table 2:** Best- and worst-performing artifact classes based on Dice scores in the independent test evaluation.

Performance	Class Name	Dice Score
High	G2_cone_cut	0.4203
High	G2_glare	0.3076
High	G1_peeling_at_edge	0.2209
High	G1_scratch_crack	0.1954
High	G2_distortion	0.1330
Low	G1_bite_marks	0.0000
Low	G2_angle_errors_superposition	0.0000
Low	G2_double_exposure	0.0000
Low	G3_writing_artifact	0.0000
Low	G4_dust_particles	0.0000

**Table 3:** Group-wise segmentation performance across artifact categories.

Group	Artifact Type	Dice Score
G1	Pre-exposure artifacts	0.1210
G2	Exposure-related artifacts	0.0915
G3	Post-exposure artifacts	0.0108
G4	Scanner-related artifacts	0.0003

## Data Availability

The data supporting the findings of this study are not publicly available due to ethical and privacy restrictions, but are available from the corresponding author upon reasonable request.
